# Country Physicians

**Published:** 1877-04-20

**Authors:** 


### COUNTRY PHYSICIANS.

The SOUTHERN MEDICAL RECORD is in a very large degree sustained by country physicians. While it has been and will continue to be our aim to interest and benefit all classes of the profession, we confess to a peculiar pleasure in having obtained the good will and support of our brethren of the country. Many years of our early professional life was spent in the country, and we retain a feeling recollection of the trials and responsibilities of country practice. We sympathize with these brethren, we know their wants and shall labor to make our journal more and more the favorite of the country and village doctor.

We trust that they will appreciate our efforts, and will interest themselves in extending our circulation.

We hope, also, that they will write for our journal. We know that there are among them many intelligent thinkers and workers in the profession—men whose extraordinary success in the unassisted emergencies of country practice, proves them to be possessed of good common sense and fine practical judgment. Such men must have discovered in the varied experiences of country practice, many useful facts, which, if published, would add to the stock of our knowledge and promote the progress of medical science. We hope that such will write for our journal. We want brief, pointed and practical facts. Whether they be, or be not, elegantly expressed, is not material. Valuable formulæ and reports of cases illustrating the success of new agents and principles in practice are particularly desired. W.

